# Targeting host calcium channels and viroporins: a promising strategy for SARS-CoV-2 therapy

**DOI:** 10.2217/fvl-2022-0203

**Published:** 2023-09-11

**Authors:** Mona Fani, Maryam Moossavi, Hasan Bakhshi, Abozar Nasiri Jahrodi, Mohammad Reza Khazdair, Amir Hossein Zardast, Shokouh Ghafari

**Affiliations:** ^1^Vector-borne Diseases Research Center, North Khorasan University of Medical Sciences, Bojnurd, Iran; ^2^North Khorasan University of Medical Sciences, Bojnurd, Iran; ^3^Department of Immunology, Birjand University of Medical Sciences, Birjand, Iran; ^4^Pharmaceutical Science & Clinical Physiology, Cardiovascular Diseases Research Center, Birjand University of Medical Sciences, Birjand, Iran; ^5^Mashhad University of Medical Sciences, Mashhad, Iran; ^6^Cellular & Molecular Research Center, Faculty of Medicine, Shahrekord University of Medical Sciences, Shahrekord, 8815713471, Iran; ^7^Department of Microbiology & Immunology, School of Medicine, Shahrekord University of Medical Sciences, Shahrekord, 8815713471, Iran

**Keywords:** calcium channel blocker, COVID-19, SARS-CoV-2, viroporins

## Abstract

Despite passing the pandemic phase of the COVID-19, researchers are still investigating various drugs. Previous evidence suggests that blocking the calcium channels may be a suitable treatment option. Ca^2+^ is required to enhance the fusion process of Severe acute respiratory syndrome coronavirus 2 (SARS-CoV-2). Also, some important inflammatory factors during SARS-CoV-2 infection are dependent on Ca^2+^ level. On the other hand, viroporins have emerged as attractive targets for antiviral therapy due to their essential role in viral replication and pathogenesis. By inhibiting the host calcium channels and viroporins, it is possible to limit the spread of infection. Therefore, calcium channel blockers (CCBs) and drugs targeting Viroporins can be considered an effective option in the fight against SARS-CoV-2.

## The pathogenesis of SARS-CoV-2

Since the last two decades, human beta-coronaviruses (ß-CoVs) have been the main topic of scientific societies and global health institutions. These zoonoses pathogens can infect the respiratory system of humans, especially in patients with underlying diseases [1,2].

Most CoVs can cause mild cold-like symptoms. SARS-CoV-2 causes COVID-19 and is associated with a wide clinical spectrum including fever, cough, dyspnea, multiple organ failure and death [3,4].

ACE-2, which belongs to the renin-angiotensin system (RAS), is expressed in various cells such as lungs, heart, hepatocytes, kidneys and endothelial cells [2]. The RAS system has two inflammatory and anti-inflammatory arms, which are controlled by ACE-1 and ACE-2, respectively; ACE-1 plays a role in causing fibrosis and vasoconstriction, and ACE-2 is an anti-coagulation enzyme that causes vasodilation [5]. SARS-CoV-2 contains a positive RNA strand and 4 different proteins including envelope (E), spike protein (S), membrane (M) and nucleoside (N). S protein binds to ACE-2 to facilitate the entry of this virus into the cell. The spike protein plays a critical role in the initiation of pathogenesis, containing a key region, receptor binding domain (RBD), with a high binding affinity to the human ACE-2 receptors. Calcium (Ca^2+^) modulates the interaction of SARS-CoV-2 S protein with human ACE-2. The sensitivity of SARS-CoV-2 spike protein to Ca^2+^ increased with the emergence of each new variant compared with the previous one and the wild-type [6].

SARS-CoV-2 infection poses therapeutic dilemmas because some drugs seem to be problematic [7,8]. Generally, some of these antiviral drugs can target DNA and RNA viruses, for example, immunomodulatory drugs inhibit the viral infection via suppression of acute inflammatory responses. The two antimalarial drugs chloroquine and hydroxychloroquine have many adverse effects, but laboratory tests have proven their antiviral activity [9]. Although there are different approved vaccines, due to the emergence of new features and different variants of this virus, there is still a need to introduce treatment methods in response to emerging virus variants [7].

Since angiotensin-converting enzyme inhibitors (ACEI) and angiotensin II receptor blockers (ARBs) can facilitate viral entry into cells by increasing ACE2 expression, there is serious debate about whether ARBs and ACEI cause accelerated disease progression or not [7].

Several vaccines have been introduced to treat COVID-19, but due to the emergence of new variants of SARS-CoV-2, the updating of vaccines and the development of parallel drugs are also important in the response to SARS-CoV-2. Production and evaluation of new drugs normally take several years; therefore, prescribing pre-existing drugs for pathogens that infect similar tissues and organs is one of the promising strategies against emerging diseases. Since retrospective observational trials and clinical studies suggest that channel blockers (CCBs) inhibit viral replication by reducing Ca^2+^ ion levels, this review introduces CCBs as therapeutic options for SARS-CoV-2 infection. Also, viroporins, virus-encoded ion channels, are key factors that take part in the viral life cycle and induction apoptosis, so they can be a promising target for the development of antiviral drugs.

Further research and drug development in these areas have significant potential for combating viral diseases. In this review, we discuss a promising and robust strategy to control SARS-CoV-2 infection.

## Ca^+2^ is an important mediator

**Figure 1.**
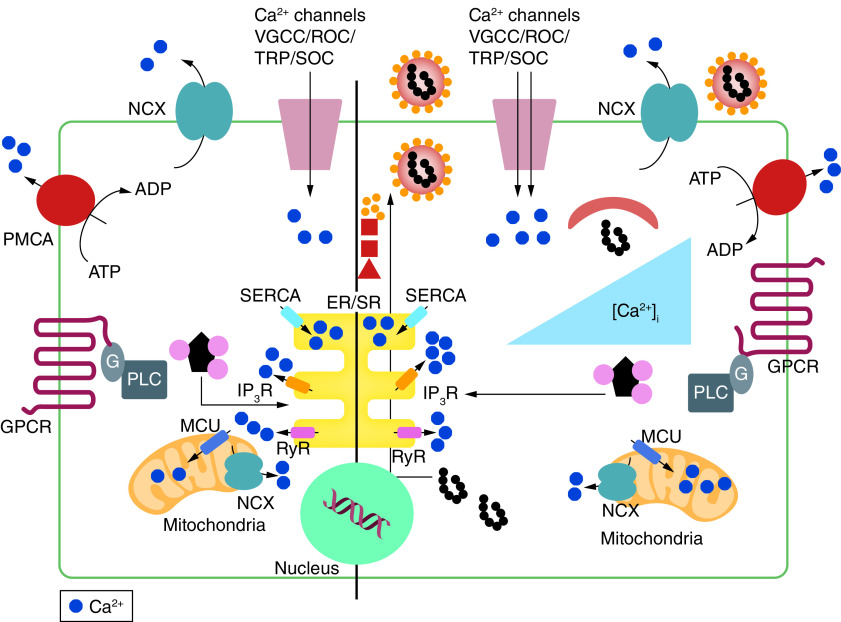
Increased cytoplasmic calcium concentration induced by viral infection in normal cells (left) and infected cells (right). Adapted with permission from [13].

In mammalian cells, Ca^+2^ is one of the intracellular regulatory molecules that can mediate important biological processes [10]. On the other hand, Ca^+2^ is responsible for important processes such as cardiovascular contractions, fertilization, metabolism, proliferation and apoptosis [10]. The endoplasmic reticulum (ER) and sarcoplasmic reticulum (SR) are two important sources of intracellular Ca^+2^. Moreover, the concentration gradient of Ca^+2^ between the intracellular sources and extracellular environment maintain via a series of channels, transporters and pumps as follows:

Voltage-gated calcium channels (VGCC), receptor-operated channels (ROC), transient receptor potential (TRP), store-operated channels (SOC), Ca^+2^ release and activated channel (CRAC channel), Ryanodine receptor (RyR), inositol-1, 4, 5-triphosphate receptor (IP3R), and calcium uniporter complex (MCUC) (Figure 1) [11,12].

In addition to the fact that Ca^+2^ controls many cellular activities, it accelerates the replication and initiation of the pathogenicity of viral infection [14]. Through three central pathways, viruses can disrupt the intracellular Ca^+2^ homeostasis: viral proteins can alter Ca^+2^ channels and the permeability of the cell membrane; also, viral proteins by banding to Ca^+2^ can destroy the stability and integrity of cell function; Ca^+2^-regulated host proteins and pathways play a critical role in viral replication and entry. Therefore, viruses cause persistent infection by hijacking Ca^+2^-dependent signaling cascades [13].

## Ca^2+^ mediates the different viral infections

Viruses replicate and establish persistent infection by employing the calcium-signaling components in several pathways: cytosolic calcium concentration is increased during viral infections (HIV-1, HTLV-1, HCV, and HHV-8) to activate calcium-dependent enzymes and transcriptional factors for viral replication and establishment of the persistent infection. The release of Ca^+2^ from the ER to the mitochondria increases the production of ATP and energy to support viral replication (HCV, CMV). Depletion of Ca^+2^ in the ER and Golgi apparatus can block host immune and protein trafficking signals, leading to evasion of the immune response. For example, some viruses (HIV-1, HCV and HBV) can stimulate apoptotic cell death to facilitate virion release. On the other hand, some viruses such as coxsackievirus and CMV inhibit apoptosis proteins and consequently immune system clearance to improve viral replication.

Several viruses, which we will discuss further, mediate the fusion between the viral and cell membranes through calcium signal transduction pathways [15]:

▪ Ebola virus (EBOV): The entry of the EBOV virus into cells is facilitated by the formation of ionic bonds between Ca^2+^ and two negatively charged residues (D522 and E540) in the fusion peptide [16].
▪ Rota virus (RV): RV infection via increasing ER permeability causes a reduction in ER Ca^2+^ stores and stimulates Ca^2+^ channels in the plasma membrane. Stromal membrane protein-interacting molecule 1 (STIM1) which spans the ER is sensitive to the reduction of ER Ca^2+^ levels. NSP4 RV mediates the depletion of ER calcium and causes the entry of stored calcium (SOCE) into the cytosol [17].
▪ Dengue virus (DENV) and West Nile virus (WNV): DENV and WNV can disturb Ca^2+^ homeostasis to replicate viral particles, as Ca^2+^-treated cells obviously showed a decrease in the generation of viral particles [18,19].
▪ Hepatitis C virus (HCV): HCV can cause ER Ca^+2^ depletion and increased Ca^+2^ uptake by mitochondria, which leads to dysfunction of ER and mitochondrial and apoptosis [20].
▪ Influenza A virus (IAV) and Human Immune deficiency (HIV) viruses: During the fusion event, Ca^+2^ cannot bind to the fusion peptide of IAV or HIV-1. The fusion peptide of influenza does not require Ca^+2^ for the fusion process, indicating that Ca^+2^ is not a booster of viral entry into target cells. In the case of HIV infection, the Ca^+2^ changes the conformation of the fusion peptide to an antiparallel β-sheet and facilitates fusion, but the absence of Ca^+2^ leads to the formation of α-helix in the fusion peptide structure [21,22].

## The role of Ca^+2^ in Betacoronaviruses (ß-CoVs) infection

ß-CoVs (MERS-CoV/SARS-CoV-1/SARS-CoV-2): Middle East respiratory syndrome (MERS), SARS-CoV-1 and SARS-CoV-2 enter the host cell by fusion. The dipeptidyl peptidase 4 (DPP4) and ACE-2 are believed to act as receptors for MERS-CoV and SARS-CoV, respectively. The ß-CoVs spike (S) protein attaches to the DPP4 (in MERS-CoV) and ACE-2 (in SARS-CoV-1 and SARS-CoV-2) to facilitate the fusion process [23]. Moreover, transmembrane protease serine 2 (TMPRSS2) as S glycoprotein priming can allow fusion with the host cell membrane. The cleavage sites are located at the border of S1 and S2 (S1/S2) and the neighborhood of the fusion peptide (S2 domain) and improve membrane fusion and viral infectivity [23]. After the first cleavage in the S1/S2 site, the S2 subunit opens for further proteolytic processing to mediate the membrane fusion [24]. During ß-CoVs infection, attachment to the host cell is mediated by proteolytic enzymes; therefore, the fusion process does not depend on pH change [25].

According to previous findings, Ca^+2^ can bind to the negatively charged amino acids aspartic acid and glutamic acid that are present in SARS-CoV-1 and MERS-CoV fusion peptides to facilitate the entry process [26]. Therefore, the depletion in extracellular and intracellular Ca^+2^ reserves leads to a decrease in the infectivity of SARS-CoV-1 and MERS-CoV particles, which shows that Ca^2+^ mediates the entry and initiation of pathogenesis in these viruses. Although there is not sufficient experimental data on the requirement of SARS-CoV-2 to Ca^+2^, the biophysical studies and sequence similarity of fusion domains of SARS-CoV-1 and SARS-CoV-2 strongly suggest that SARS-CoV-2 is dependent on Ca^+2^ for fusion and entry process [27,28]. Singh *et al.* reported that the sensitivity of SARS-CoV-2 spike protein to Ca^+2^ was higher in D614G, beta, alpha and delta variants than in wild-type, respectively. The affinity of beta S protein to ACE2 is 15% lower than the alpha due to additional mutations (K417N and E484K). Generally, the S protein in every variant is more sensitive to Ca^2+^ concentration compared with the Wuhan strain, suggesting that mutations in the S protein stimulate more exposure of the RBD to the ACE-2 receptors and higher fusion activity. Therefore, it is likely that the high infectivity and transmissibility of the delta variant compared with other variants is due to the increase in the affinity of protein S to Ca^2+^ [6]. The dependency of ß-CoVs on Ca^+2^ is also interesting from another point of view; ß-CoVs S protein activates the Ca^2+^ channels in the host cell, leading to the production and subsequent secretion of a lipid coat, resulting in the fusion of the infected cell with neighboring cells that express ACE2. This complex strategy may enable the infected cell to have a longer lifespan. SARS-CoV-2 has the ability to form a similar structure with lymphocytes, which are crucial immune cells, as seen in tumor cells, causing evading SARS-CoV-2 from the host immune system [28,29].

## Calcium pumps & channels

Extracellular medium and intracellular organelles such as endoplasmic reticulum and sarcoplasmic reticulum are the two main sources of cellular Ca^2+^ [13]. A number of Ca^2+^ channels are on the cell plasma membrane and play a role in the entry of Ca^2+^ into the intracellular environment. These channels include voltage-gated calcium channels (VGCC) that are activated by membrane depolarization, receptor-operated calcium channels (ROCC) that are activated by extracellular agonists or intracellular signals and store-operated calcium channels (SOCC) which are activated by depletion of internal Ca^2+^ stores and transient receptor potential channels (TRPC) which are activated by chemical, thermal and mechanical stimulants.

The IP3 receptor (IP3R) and the ryanodine receptors (RyR) are located on the internal organelles and stimulate or inhibit the Ca^2+^ release from them into the cytosol. In addition to the calcium channel, the plasma membrane also contains Ca^2+^ pumps such as the plasma membrane Ca^2+^-ATPase (PMCA), sarco/endoplasmic reticulum Ca^2+^-ATPase (SERCA), and a Na^+^/Ca^2+^ exchanger(NCX) which transfer Ca^2+^ from the cytosol to the extracellular environment or to intracellular stores [13].

## The importance of calcium channel proteins in viral infections

Generally, viral infections modulate the activity of these channels and pumps in response to infection to provide a suitable environment for the virus life cycle. By increasing the concentration of cytosolic Ca^2+^, viruses activate Ca^2+^-dependent enzymes and transcription factors for further virus replication. The increased Ca^2+^ in the mitochondria results in generating more energy and can help the viral continuous replication. Also, increased Ca^2+^ concentrations in the ER or Golgi may promote increased expression of viral proteins by inhibiting host protein trafficking. Viruses by disrupting the Ca^2+^ channels can suppress the activity of T cells, anti-apoptotic proteins and other preventive factors [13].

The VGCC channel is a structural complex consisting of five different subunits including α1, α2, β, δ and γ that are found in 4 different types L, N, P and T. L-type is located in the cell membrane of skeletal, cardiac and smooth muscles, and causes the contraction of muscle cells [30]. L-type is located in the cell membrane of skeletal, cardiac and smooth muscles and causes contraction of muscle cells. T-type channels are found in the cardiovascular and renal systems. N-type and P-type are located on neurons and Purkinje cells, respectively [31].

SOOC channels are another Ca^2+^ channels found in non-excitable cells. Tow molecules of the Orai1 and STIM1 are located on the plasma and ER membranes, respectively. Depletion of ER Ca^2+^ stores induces the interaction of STIM1 with Orai1, leading to SOOC activation for Ca^2+^ entry [32]. SOCC is involved in the assembly and release of viruses, on the other hand, an increase in cytosolic Ca^+2^ has been demonstrated in many infections caused by enveloped viruses. For example, Rotavirus infection increases the SOCC permeability to cytosolic Ca^2+^ for virion maturation. Rotavirus NSP4 is an important factor in the depletion of ER Ca^2+^ by activation of STIM1 and ORAI, indicating that inhibition of STIM1 and ORAI can inhibit the rotavirus infection [14,33]. The importance of other channels in the pathogenesis of viral infections is also known [13].

The role of Ca^2+^ in increasing the infectivity of viruses, including coronaviruses, has been proven. Coronaviruses envelope (E) protein disrupts Ca^2+^ homeostasis by forming a Ca^2+^ channel in the membranes of Golgi and endoplasmic reticulum, which leads to the activation of the inflammatory factor NLRP3 and pro-inflammatory cytokines, which leads to olfactory and neurological problems in COVID-19 patients [13]. Considering the importance of the presence of Ca^+2^ in the entry of these viruses, investigating the therapeutic potential of CCB drugs against SARS-CoV-2 is one of the topics under discussion.

## Classification of CCBs

CCBs which are Ca^2+^ antagonists and comprised of a heterogeneous group of chemical structures, prevent the entry of extracellular and intracellular sources of calcium into the cytoplasm of excitable cells such as skeletal, cardiac, and smooth muscle cells, leading to relaxation, vasodilation and lowering of blood pressure. CCBs which are used to treat a wide range of medical conditions including angina pectoris, chest pain, high blood pressure, rapid and sudden heartbeats, subarachnoid hemorrhage and pulmonary hypertension, migraines and inhibition of uterine contractions [34].

CCBs that block Ca^2+^ channels are divided into dihydropyridines and non-dihydropyridines groups [35].

1) Dihydropyridines include amlodipine, clevidipine, felodipine, isradipine, lercanidipine, nicardipine, nifedipine, nimodipine and nisoldipine; 2) non-dihydropyridines include Benzothiazepines (diltiazem) and Phenylalkylamines (verapamil). Dihydropyridine drugs cause dilation of arterial vessels by directly affecting vascular smooth muscles. Also, these drugs have moderate selectivity for vascular calcium channels, while non-dihydropyridine drugs act on the myocardium, causing a decrease in heart rate and contractility, as well as a slight increase in arterial dilation. Non-dihydropyridines have moderate to low selectivity for calcium channels [36].

## CCB Drugs against viral infections

A number of VGCC blockers are currently FDA-licensed, and research into other blockers is ongoing. VGCC are the most prevalent calcium channel expressed in a variety of host cells. The first known VGCC blocker is Verapamil which can prevent influenza A virus (IAV) infection in the fusion step. IAV increases the cytosolic concentration of Ca^+2^ by the disturbance of Ca^+2^ channels to facilitate the entry process into the cell [32,35]. *In* *vitro* and *in* *vivo* studies show that the IAV hemagglutinin (HA) protein attaches to sialic acids and also the VGCC channel, because the mutation in the VGCC channel caused weak binding of the IAV to target cells, indicating that the VGCC channel can be a host cell receptor for IAV attachment and fusion process. Also, IAV-infected Mice that were treated with diltiazem lived longer than other mice [37].

The gp120 and transcription activator (Tat) proteins of HIV, by down-regulating the VGCC activity, increase cytosolic Ca^+2^ and facilitate the efficient replication of this virus. Therefore, gp120- and Tat-blocking drugs are potential candidates for HIV-1 therapy due to maintaining intracellular Ca^+2^ stability [38]. Rotavirus infection increases the permeability of VOC and ROC channels to Ca^+2^ entry to produce mature viral particles. Verapamil partially prevents the permeability of these channels to Ca^+2^ [32].

The antiviral effect of CCBs drugs has been proven in many other studies:

- Nifedipine and verapamil inhibit the increase of Ca^+2^ in the cytosol of immune cells infected with herpes viruses such as Epstein–Barr virus (EBV) and human cytomegalovirus (CMV) [32].
- Verapamil has also been shown to inhibit the permeability of Ca^+2^ and K^+^ ion channels in simian virus 40 (SV40) infection [13]. Tetrandrine, a two-pore channel (TPC) blocker, inhibits the uncoating process in SV40 and Merkel cell polyomavirus (MCPyV) infections [39,40].
- Benidipine hydrochloride, cilnidipine, and manidipine can inhibit Japanese encephalitis virus (JEV) [41], DENV, WNV and Zika virus infections at the replication step [42].

In vivo studies show that diltiazem, nimodipine, tetrandrine and verapamil inhibit EBOV-induced viral infection by blocking VGCC channels. Also, *in vitro* and *in vivo* studies confirm that memantine can prevent neuronal cell apoptosis caused by Zika virus infection. Gabapentin also acts as an L-type channel blocker in arenavirus hemorrhagic fever [13].

In addition, according to previous studies, verapamil has an inhibitory effect on other viral infections such as CMV, mouse mammary tumor virus, human rhinovirus 2, Sindebis, vesicular stomatitis virus (VZV), measles and vaccinia viruses [43].

## CCBs drugs against coronaviruses

The utilization of Ca^+2^ by various viruses is a common pathway for fusion, entry and viral replication in host cells. In fact, Ca^+2^ mediates the interaction between viral attachment protein and host cell receptor to facilitate the virus entry [44].

Previously, it has been shown that antihypertensive drugs nifedipine and amlodipine are suitable therapeutic options due to their ability to inhibition of viral entry into lung epithelial cells [44–46]. Also, nifedipine and amlodipine are used in the treatment of various pulmonary and cardiovascular diseases.

In vitro studies show that the elevated cytosolic Ca^+2^ stimulates the expression of reactive oxygen species (ROS) and subsequently apoptosis in cells. In cells under oxidative stress, expression of nuclear factor erythroid-2-related factor 2 (Nrf2) increases and migrates to the nucleus to activate antioxidant enzymes. Nifedipine, a suitable drug for reducing pulmonary blood pressure in the clinic, can activate Nrf2 and inhibit the NFkB pathway, reducing oxidative stress, cell death, nuclear condensation and cytosolic Ca^+2^ [45].

On the other hand, patients with ß-CoV suffer from vasoconstriction and hypertension. In addition, TPC plays an important role in the entry of SARS-CoV into the cell, indicating that inhibition of TPC is one of the appropriate targets in the development of antiviral drugs, as Ou *et al.* showed that Tetrandrine can target TPC to block the entry process of SARS-CoV-2 [47].

## In vivo studies evaluated the CCB effects on SARS-CoV-2

Retrospective studies have specifically investigated the efficacy of CCBs drugs against COVID-19 disease. Zhang *et al.* studied 90 hypertensive COVID-19 patients and showed that nifedipine and amlodipine have significant antiviral effects [48].

A clinical study showed that amlodipine reduced mortality in hypertensive patients with SARS CoV-2 who were treated with amlodipine than in the non-amlodipine group, this finding provides evidence for the treatment of hypertensive COVID-19 patients. In a parallel study, Solaimanzadeh *et al.* 65 COVID-19 patients with high blood pressure divided into two groups of 24 people (under treatment with amlodipine or nifedipine) and 41 people (under treatment with non-CCBs drugs). The results of this group confirmed that the mortality rate of treatment with amlodipine or nifedipine is significantly lower [49]. However, CCBs in the study of Reynolds *et al.* were associated with an unfavorable outcome, analysis of this group's study showed that probably previous use of CCBs are associated with a relatively severe disease [50].

## In vitro studies evaluated the CCB effects on SARS-CoV-2

During the SARS-CoV-2 pandemic, some clinical drugs were studied to see if they could inhibit the replication of SARS-CoV-2 *in vitro* as CCBs. The findings of these studies confirmed that the anti-SARS-CoV-2 effect of CCBs is correlated with reducing intracellular calcium levels. Zhang *et al.* showed that Vero E6 cells infected with SARS-CoV-2 treated with amlodipine or benidipine have significant antiviral effects against infection compared with ARBs or ACEIs that had no inhibitory effect on SARS-CoV-2 [48].

In a parallel study, Strauss *et al.* showed that treatment of SARS-CoV-2-infected Vero E6 cells with US FDA-approved CCBs including amlodipine, felodipine and nifedipine could severely limit SARS-CoV-2 entry, proliferation and infection with minimal toxicity for lung cells [51]. Interestingly, Hoagland *et al.* showed that Amlodipine-treated SARS-CoV-2 infected A549-ACE2 cells enhance the type-1 interferon expression *in*- *vitro* that has a critical role against SARS-CoV-2 infection [52].

## Viroporin in viral infections can be a new therapeutic target

Viroporins have been known as virally encoded hydrophobic proteins, which oligomerize within the host cell's membrane, thereby inducing the creation of hydrophilic pores. This particular activity results in the alteration of numerous cellular functions such as membrane permeability, Ca^2+^ homeostasis, membrane remodeling and glycoprotein trafficking. Also, one of the main functions of viroporins is to facilitate the assembly and release of viral particles from infected cells. Furthermore, the ion channel activity of viroporins holds the potential to disrupt the homeostasis of intracellular ions such as Na^+^, K^+^, Ca^2+^ and Cl^-^ [53].

A substantial portion of the viroporins are distinctly localized on intracellular organelles such as the Golgi and the endoplasmic reticulum (ER), while only a limited number are identified on the plasma membrane.

The functional activities of viroporins can significantly disrupt the signaling pathways such as autophagy, apoptosis and cellular immune responses to promote virus replication in the host cell, indicating the pathological feature of viroporins can be independent of viral infection. Knockout of viroporins genes from the viral genome can reduce viral pathogenicity, indicating the critical role of these proteins in the life cycle of viruses. Thus, viroporins can be a suitable option for designing antiviral drugs [54].

Viroporins have been identified in several RNA viruses, but we are exclusively discussing coronaviruses, especially SARS-CoV-2. The order of SARS-CoV-2 is RNA 5′-replicate (ORF1a/b)-S-E-M-N-poly (A)-3′, furthermore at its 3′ portion there is several ORFs including ORF3a, ORF3b, ORF6, ORF7a, ORF7b, ORF8, ORF9b, ORF9c and ORF10. Among them, E, ORF3a and ORF8a are viroporin [5].

Although the genomic similarity between ORF3a protein sequences of SARS-CoV and SARS-CoV-2 is 72.4%, the E protein between the two viruses shows high conservation with 94.7% identity [54]. Due to the higher ionic permeability of the SARS-CoV E protein compared with orf3a and orf8a, the E protein may play a more important role in the infectivity of this virus. Due to the high similarity of the E protein in SARS-CoV-1 and SARS-CoV-2, this protein is expected to have a similar role in both viruses [55,56].

Deletion of the *E* gene from SARS-CoV-1 and MERS-CoV in infected animal models produced attenuated viral particles, introducing a new solution to protect against SARS-CoV. E protein stimulates the NF-κB inflammatory pathway and activates p38 MAPK, which subsequently induces the production of proinflammatory cytokine and cytokine storm, leading to exacerbation of immunopathology and widespread lung injury. In line with this result, mice infected with E SARS-CoV-1 showed acute respiratory distress syndrome (ARDS) and pulmonary edema.

The presence of E protein was observed to cause an increase in both edema and a proinflammatory response mediated by interleukin 1 beta (IL-1β) within the lung parenchyma [57].

IL-1β is a potent proinflammatory cytokine that is overexpressed in ARDS. Briefly, Coronaviruses E protein disrupts ion homeostasis (especially Ca^2+^) by the formation of ion channels in the ER and Golgi membranes. This stimulates the production of the inflammatory protein NLRP3 and pro-inflammatory cytokines IL-1β, as observed in respiratory infection and degenerative diseases caused by coronaviruses [58–60].

Also, some studies show that the neurological and olfactory disorders observed in COVID-19 patients can be caused by the function of E protein. CNS-resident microglia and astrocytes play a critical role in homeostasis and neuroinflammation. Animal studies show that these cells are activated by injection of E protein and release pro-inflammatory cytokines such as IL-1β and IL-6. In addition to these cytokines being related to the apoptosis of neural cells, they can increase the permeability of the blood-brain barrier and the infiltration of immune cells into the brain via the degradation of tight junctions, which provides an opportunity for the entrance of the other infectious agents and cytokines to the brain, leading to the neurological symptoms [61].

The importance of astrocytes is also significant in other aspects; Human cellular cultured astrocytes infected with SARS-CoV-2 show increased infection and proliferation compared with other cells. Also, in post-mortem studies, SARS-CoV-2 nucleocapsid was found more often in these cells. On the other hand, due to the greater vulnerability of elderly patients, the hypothesis that is proposed is that the aging of astrocytes may make these people susceptible to the dysfunction caused by SARS-CoV-2 [61].

Since the deletion of the *E* gene leads to the weakening of the replication of SARS-CoV-2, it can be a promising and potential target for the development of antiviral drugs. Also, *in* *vivo* studies show that two drugs, amantadine and hexamethylene amiloride, can inhibit SARS-CoV-2 virus viroporin [53].

## Conclusion

This review summarizes the disruption of the intracellular calcium signaling system and homeostasis of Ca^+2^ during viral infection leading to increased viral replication. Although the effect of viruses on the host intracellular Ca^+2^ environment is complicated, the drugs targeting the calcium channel are potential therapeutic options to treat viral infections. Meanwhile, our knowledge of the molecular aspects of calcium-virus interaction is scanty and requires further investigations and evidence to confirm whether CCBs can be an effective treatment for SARS-CoV-2 infection. Studies on antiviral drugs show that CCBs, especially the L-type, such as felodipine and nifedipine, inhibit the level of virus entry, so there is almost no pathological effect caused by the virus in infected subjects. There is also accumulated evidence that viroporins are involved in the disruption of Ca^+2^ hemostasis, result in neuropsychiatric disorders in COVID-19 patients. Therefore, pharmacological inhibition of these viral proteins may also have therapeutic value, suggesting viroporin-defective viruses can be attenuated vaccine candidates.

## Future perspective

There are concerns about the emergence of viral infection in the future which could increase the fatality rate. As many viruses modulate the Ca^2+^ signaling to replicate their genomes, CCBs drugs can be potential therapeutic options to treat viral infections. On the other hand, viroporins that are encoded by many viruses can be one the best candidates for vaccines and drugs.

**Executive summary**

- This review tried to propose a promising and robust strategy to control SARS-CoV-2 infection.

**The pathogenesis of SARS-CoV-2**

- The S protein plays a critical role in the initiation of SARS-CoV-2 pathogenesis. The sensitivity of SARS-CoV-2 spike protein to Ca^2+^ increased with the emergence of each new variant compared with the previous one and the wild-type.

**Ca^+2^ is an important mediator**

- Viruses can disrupt intracellular Ca^+2^ homeostasis by enhancing the permeability of the cell membrane; destroying the integrity of cell function; hijacking Ca^+2^-dependent signaling cascades.

**Ca^2+^ mediates the different viral infections**

- Viruses replicate and establish persistent infection by employing the calcium-signaling components in several pathways.

**The role of Ca^+2^ in betacoronaviruses (ß-CoVs) infection**

- The depletion in extracellular and intracellular Ca^+2^ reserves leads to a decrease in the infectivity of SARS-CoV-1 and MERS-CoV particles, which shows that Ca^2+^ mediates the entry and initiation of pathogenesis in these viruses.

**Calcium pumps & channels**

- Extracellular medium, intracellular organelles and cell plasma membrane play a role in the entry of Ca^2+^ into the intracellular environment.

**The importance of calcium channel proteins in viral infections**

- By increasing the concentration of cytosolic Ca^2+^, viruses activate Ca^2+^-dependent enzymes and transcription factors for further virus replication. The increased Ca^2+^ in the mitochondria results in generating more energy and can help the viral continuous replication. Also, increased Ca^2+^ concentrations in the ER or Golgi may promote increased expression of viral proteins by inhibiting host protein trafficking. Viruses by disrupting the Ca^2+^ channels can suppress the activity of T cells, anti-apoptotic proteins and other preventive factors.

**Classification of calcium channel blockerss**

- Calcium channel blockers (CCBs) that block Ca^2+^ channels are divided into dihydropyridines and non-dihydropyridines groups.

**CCB drugs against viral infections**

- A number of VGCC blockers are currently FDA-licensed, and research into other blockers is ongoing. The first known VGCC blocker is Verapamil which can prevent influenza A virus (IAV) infection in the fusion step.

**CCB drugs against coronaviruses**

- Ca^+2^ mediates the interaction between viral attachment protein and host cell receptor to facilitate the virus entry.

**Viroporin in viral infections can be a new therapeutic target**

- Viroporins have been known as virally encoded hydrophobic proteins, which oligomerize within the host cell's membrane, thereby inducing the creation of hydrophilic pores. This particular activity results in the alteration of numerous cellular functions such as membrane permeability, Ca^2+^ homeostasis, membrane remodeling and glycoprotein trafficking.

## References

[1] Su S, Wong G, Shi W Epidemiology, genetic recombination, and pathogenesis of coronaviruses. Trends Microbiol. 24(6), 490–502 (2016). 27012512 10.1016/j.tim.2016.03.003 PMC7125511

[2] Fattahi Z, Mohseni M, Jalalvand K SARS-CoV-2 outbreak in Iran: the dynamics of the epidemic and evidence on two independent introductions. Transbound. Emerg. Diseas. 69(3), 1375–1386 (2022). 10.1111/tbed.14104 PMC8251331 33835709

[3] Ebrahimi S, Khanbabaei H, Abbasi S CRISPR-Cas system: a promising diagnostic tool for COVID-19. Avicenna J. Med. Biotechnol. 14(1), 3 (2022). 35509363 10.18502/ajmb.v14i1.8165 PMC9017467

[4] Zandi M, Soltani S, Fani M, Abbasi S, Ebrahimi S, Ramezani A. Severe acute respiratory syndrome coronavirus 2 and respiratory syncytial virus coinfection in children. Osong Public Health Res. Perspect. 12(5), 286 (2021). 34719220 10.24171/j.phrp.2021.0140 PMC8561020

[5] Zandi M, Shafaati M, Kalantar-Neyestanaki D The role of SARS-CoV-2 accessory proteins in immune evasion. Biomed. Pharmacother. 156, 113889 (2022). 36265309 10.1016/j.biopha.2022.113889 PMC9574935

[6] Singh P, Mukherji S, Basak S, Hoffmann M, Das DK. Dynamic Ca^2+^ sensitivity stimulates the evolved SARS-CoV-2 spike strain-mediated membrane fusion for enhanced entry. Cell Reports 39(3), (2022). 10.1016/j.celrep.2022.110694 PMC8993541 35397208

[7] Aronson JK, Ferner RE. Drugs and the renin-angiotensin system in COVID-19. BMJ 369, m1313 (2020). 32241880 10.1136/bmj.m1313

[8] Khazdair MR, Ghafari S, Sadeghi M. Possible therapeutic effects of Nigella sativa and its thymoquinone on COVID-19. Pharmaceut. Biol. 59(1), 694–701 (2021). 10.1080/13880209.2021.1931353 PMC8204995 34110959

[9] Yao X, Ye F, Zhang M *In vitro* antiviral activity and projection of optimized dosing design of hydroxychloroquine for the treatment of severe acute respiratory syndrome coronavirus 2 (SARS-CoV-2). Clin. Infect. Dis. 71(15), 732–739 (2020). 32150618 10.1093/cid/ciaa237 PMC7108130

[10] Berridge MJ, Bootman MD, Roderick HL. Calcium signalling: dynamics, homeostasis and remodelling. Nat. Rev. Mol. Cell Biol. 4(7), 517–529 (2003). 12838335 10.1038/nrm1155

[11] De Stefani D, Rizzuto R, Pozzan T. Enjoy the trip: calcium in mitochondria back and forth. Annu. Rev. Biochem. 85, 161–192 (2016). 27145841 10.1146/annurev-biochem-060614-034216

[12] Hogan PG, Rao A. Store-operated calcium entry: mechanisms and modulation. Biochem. Biophys. Res. Commun. 460(1), 40–49 (2015). 25998732 10.1016/j.bbrc.2015.02.110 PMC4441756

[13] Chen X, Cao R, Zhong W. Host calcium channels and pumps in viral infections. Cells 9(1), 94 (2019). 31905994 10.3390/cells9010094 PMC7016755

[14] Clark KB, Eisenstein EM. Targeting host store-operated Ca^2+^ release to attenuate viral infections. Curr. Topics Medicin. Chem. 13(16), 1916–1932 (2013). 10.2174/15680266113139990128 23895094

[15] Dubé M, Rey FA, Kielian M. Rubella virus: first calcium-requiring viral fusion protein. PLOS Pathog. 10(12), e1004530 (2014). 25474548 10.1371/journal.ppat.1004530 PMC4256232

[16] Nathan L, Lai AL, Millet JK Calcium ions directly interact with the Ebola virus fusion peptide to promote structure-function changes that enhance infection. ACS Infect. Dis. 6(2), 250–260 (2019). 31746195 10.1021/acsinfecdis.9b00296 PMC7040957

[17] Hyser JM, Utama B, Crawford SE, Broughman JR, Estes MK. Activation of the endoplasmic reticulum calcium sensor STIM1 and store-operated calcium entry by rotavirus requires NSP4 viroporin activity. J. Virol. 87(24), 13579–13588 (2013). 24109210 10.1128/JVI.02629-13 PMC3838237

[18] Dionicio CL, Pena F, Constantino-Jonapa LA Dengue virus induced changes in Ca^2+^ homeostasis in human hepatic cells that favor the viral replicative cycle. Virus Res. 245, 17–28 (2018). 29269104 10.1016/j.virusres.2017.11.029

[19] Scherbik SV, Brinton MA. Virus-induced Ca^2+^ influx extends survival of west nile virus-infected cells. J. Virol. 84(17), 8721–8731 (2010). 20538858 10.1128/JVI.00144-10 PMC2918993

[20] Benali-Furet NL, Chami M, Houel L Hepatitis C virus core triggers apoptosis in liver cells by inducing ER stress and ER calcium depletion. Oncogene 24(31), 4921–4933 (2005). 15897896 10.1038/sj.onc.1208673

[21] Lai AL, Millet JK, Daniel S, Freed JH, Whittaker GR. The SARS-CoV fusion peptide forms an extended bipartite fusion platform that perturbs membrane order in a calcium-dependent manner. J. Mol. Biol. 429(24), 3875–3892 (2017). 29056462 10.1016/j.jmb.2017.10.017 PMC5705393

[22] Nieva JL, Nir S, Muga A, Goni FM, Wilschut J. Interaction of the HIV-1 fusion peptide with phospholipid vesicles: different structural requirements for fusion and leakage. Biochemistry 33(11), 3201–3209 (1994). 8136355 10.1021/bi00177a009

[23] Fani M, Teimoori A, Ghafari S. Comparison of the COVID-2019 (SARS-CoV-2) pathogenesis with SARS-CoV and MERS-CoV infections. Fut. Virol. 15(5), 317–323 (2020).

[24] Belouzard S, Millet JK, Licitra BN, Whittaker GR. Mechanisms of coronavirus cell entry mediated by the viral spike protein. Viruses 4(6), 1011–1033 (2012). 22816037 10.3390/v4061011 PMC3397359

[25] White JM, Whittaker GR. Fusion of enveloped viruses in endosomes. Traffic 17(6), 593–614 (2016). 26935856 10.1111/tra.12389 PMC4866878

[26] Lai AL, Freed JH. Negatively charged residues in the membrane ordering activity of SARS-CoV-1 and-2 fusion peptides. Biophys. J. 121(2), 207–227 (2022). 34929193 10.1016/j.bpj.2021.12.024 PMC8683214

[27] Straus MR, Tang T, Lai AL Ca^2+^ ions promote fusion of Middle East respiratory syndrome coronavirus with host cells and increase infectivity. J. Virol. 94(13), e00426–e00420 (2020). 32295925 10.1128/JVI.00426-20 PMC7307142

[28] Tang T, Bidon M, Jaimes JA, Whittaker GR, Daniel S. Coronavirus membrane fusion mechanism offers a potential target for antiviral development. Antiviral Res. 178, 104792 (2020). 32272173 10.1016/j.antiviral.2020.104792 PMC7194977

[29] Candido KL, Eich CR, De Fariña LO Spike protein of SARS-CoV-2 variants: a brief review and practical implications. Brazilian J. Microbiol. 53(3), 1133–1157 (2022). 10.1007/s42770-022-00743-z PMC8994061 35397075

[30] Beale JM, Block J, Hill R. Organic medicinal and pharmaceutical chemistry. Lippincott Williams & Wilkins Philadelphia (2010). http://www.thePoint.lww.com/Beale12e

[31] Atlas D. Voltage-gated calcium channels function as Ca^2+^-activated signaling receptors. Trends Biochem. Sci. 39(2), 45–52 (2014). 24388968 10.1016/j.tibs.2013.12.005

[32] Zhou Y, Frey TK, Yang JJ. Viral calciomics: interplays between Ca^2+^ and virus. Cell Calcium 46(1), 1–17 (2009). 19535138 10.1016/j.ceca.2009.05.005 PMC3449087

[33] Chen X, Cao R, Zhong W. Host calcium channels and pumps in viral infections. Cells 9(1), 94 (2019). 31905994 10.3390/cells9010094 PMC7016755

[34] Crespi B, Alcock J. Conflicts over calcium and the treatment of COVID-19. Evolut. Med. Public Health 9(1), 149–156 (2021). 10.1093/emph/eoaa046 PMC7717197 33732462

[35] Dedea L. How do dihydropyridine and nondihydropyridine CCBs differ? JAAPA 25(3), 15 (2012). 10.1097/01720610-201203000-00002 22514952

[36] Waller DG, Sampson A, Hitchings A. Medical pharmacology and therapeutics E-Book. Elsevier Health Sciences (2021).

[37] Fujioka Y, Nishide S, Ose T A sialylated voltage-dependent Ca^2+^ channel binds hemagglutinin and mediates influenza A virus entry into mammalian cells. Cell Host Microbe 23(6), 809–818; e805 (2018). 29779930 10.1016/j.chom.2018.04.015

[38] Haughey NJ, Mattson MP. Calcium dysregulation and neuronal apoptosis by the HIV-1 proteins Tat and gp120. JAIDS 31, S55–S61 (2002). 12394783 10.1097/00126334-200210012-00005

[39] Dobson SJ, Mankouri J, Whitehouse A. Identification of potassium and calcium channel inhibitors as modulators of polyomavirus endosomal trafficking. Antiviral Res. 179, 104819 (2020). 32389733 10.1016/j.antiviral.2020.104819 PMC7205714

[40] Fujioka Y, Tsuda M, Nanbo A A Ca^2+^-dependent signalling circuit regulates influenza A virus internalization and infection. Nat. Commun. 4(1), 2763 (2013). 24434940 10.1038/ncomms3763

[41] Wang S, Liu Y, Guo J Screening of FDA-approved drugs for inhibitors of Japanese encephalitis virus infection. J. Virol. 91(21), e01055–e01017 (2017). 28814523 10.1128/JVI.01055-17 PMC5640845

[42] Li H, Zhang L-K, Li S-F Calcium channel blockers reduce severe fever with thrombocytopenia syndrome virus (SFTSV) related fatality. Cell Res. 29(9), 739–753 (2019). 31444469 10.1038/s41422-019-0214-z PMC6796935

[43] Alam M, Mostafa A, Kanrai P, Müller C, Dzieciolowski J. Verapamil has antiviral activities that target different steps of the influenza virus replication cycle. J. Antivir. Antiretrovir. 8(4), 121–130 (2016).

[44] Straus MR, Bidon MK, Tang T, Jaimes JA, Whittaker GR, Daniel S. Inhibitors of L-type calcium channels show therapeutic potential for treating SARS-CoV-2 infections by preventing virus entry and spread. ACS Infect. Dis. 7(10), 2807–2815 (2021). 34498840 10.1021/acsinfecdis.1c00023

[45] Manohar K, Gupta RK, Gupta P FDA approved L-type channel blocker Nifedipine reduces cell death in hypoxic A549 cells through modulation of mitochondrial calcium and superoxide generation. Free Radic. Biol. Med. 177, 189–200 (2021). 34666149 10.1016/j.freeradbiomed.2021.08.245 PMC8520174

[46] Jayaseelan VP, Paramasivam A. Repurposing calcium channel blockers as antiviral drugs. J. Cell Commun. Signal. 14, 467–468 (2020). 32815099 10.1007/s12079-020-00579-y PMC7438026

[47] Ou X, Liu Y, Lei X Characterization of spike glycoprotein of SARS-CoV-2 on virus entry and its immune cross-reactivity with SARS-CoV. Nat. Commun. 11(1), 1620 (2020). 32221306 10.1038/s41467-020-15562-9 PMC7100515

[48] Zhang L-K, Sun Y, Zeng H Calcium channel blocker amlodipine besylate therapy is associated with reduced case fatality rate of COVID-19 patients with hypertension. Cell Discov. 6(1), 96 (2020). 33349633 10.1038/s41421-020-00235-0 PMC7752915

[49] Solaimanzadeh I. Nifedipine and amlodipine are associated with improved mortality and decreased risk for intubation and mechanical ventilation in elderly patients hospitalized for COVID-19. Cureus 12(5), (2020). 10.7759/cureus.8069 PMC7219014 32411566

[50] Reynolds HR, Adhikari S, Pulgarin C Renin-angiotensin-aldosterone system inhibitors and risk of COVID-19. N. Engl. J. Med. 382(25), 2441–2448 (2020). 32356628 10.1056/NEJMoa2008975 PMC7206932

[51] Straus MR, Bidon M, Tang T, Whittaker GR, Daniel S. FDA approved calcium channel blockers inhibit SARS-CoV-2 infectivity in epithelial lung cells. BioRxiv (2020).

[52] Hoagland DA, Clarke DJ, Møller R Modulating the transcriptional landscape of SARS-CoV-2 as an effective method for developing antiviral compounds. BioRxiv (2020).

[53] Mandala VS, Mckay MJ, Shcherbakov AA, Dregni AJ, Kolocouris A, Hong M. Structure and drug binding of the SARS-CoV-2 envelope protein transmembrane domain in lipid bilayers. Nature Struct. Mol. Biol. 27(12), 1202–1208 (2020). 33177698 10.1038/s41594-020-00536-8 PMC7718435

[54] Breitinger U, Farag NS, Sticht H, Breitinger H-G. Viroporins: structure, function, and their role in the life cycle of SARS-CoV-2. Internat. J. Biochem. Cell Biol. 145, 106185 (2022). 10.1016/j.biocel.2022.106185 PMC8868010 35219876

[55] Chen I-Y, Moriyama M, Chang M-F, Ichinohe T. Severe acute respiratory syndrome coronavirus viroporin 3a activates the NLRP3 inflammasome. Front. Microbiol. 10, 50 (2019). 30761102 10.3389/fmicb.2019.00050 PMC6361828

[56] Siu K-L, Yuen K-S, Castaño-Rodriguez C Severe acute respiratory syndrome coronavirus ORF3a protein activates the NLRP3 inflammasome by promoting TRAF3-dependent ubiquitination of ASC. FASEB J. 33(8), 8865 (2019). 31034780 10.1096/fj.201802418R PMC6662968

[57] Xu H, Akinyemi IA, Chitre SA SARS-CoV-2 viroporin encoded by ORF3a triggers the NLRP3 inflammatory pathway. Virology 568, 13–22 (2022). 35066302 10.1016/j.virol.2022.01.003 PMC8762580

[58] Potere N, Del Buono MG, Caricchio R Interleukin-1 and the NLRP3 inflammasome in COVID-19: pathogenetic and therapeutic implications. EBioMedicine 85, 104299 (2022). 36209522 10.1016/j.ebiom.2022.104299 PMC9536001

[59] Lamers MM, Haagmans BL. SARS-CoV-2 pathogenesis. Nat. Rev. Microbiol. 20(5), 270–284 (2022). 35354968 10.1038/s41579-022-00713-0

[60] Nieto-Torres JL, Verdiá-Báguena C, Jimenez-Guardeño JM Severe acute respiratory syndrome coronavirus E protein transports calcium ions and activates the NLRP3 inflammasome. Virology 485, 330–339 (2015). 26331680 10.1016/j.virol.2015.08.010 PMC4619128

[61] Gonçalves C-A, Sesterheim P, Wartchow KM, Bobermin LD, Leipnitz G, Quincozes-Santos A. Why antidiabetic drugs are potentially neuroprotective during the Sars-CoV-2 pandemic: the focus on astroglial UPR and calcium-binding proteins. Front. Cell. Neurosci. 16, 905218 (2022). 35966209 10.3389/fncel.2022.905218 PMC9374064

